# A case report on imaging findings of rare segmental necrotizing granulomatous neuritis of leprosy involving ulnar nerve

**DOI:** 10.5339/qmj.2024.36

**Published:** 2024-06-23

**Authors:** S. B. S. Netam, Nilesh Gupta, Nobal Chandrakar

**Affiliations:** 1 Department of Radiodiagnosis, Pt. J.N.M. Medical College & Associated Dr. B.R. Ambedkar Hospital, Raipur, Chhattisgarh, India; 2 Department of Radiodiagnosis, Sri Balaji Institute of Medical Sciences, Raipur, Chhattisgarh, India; 3 Department of Radiodiagnosis, Pt. J.N.M. Medical College & Associated Dr. B.R. Ambedkar Hospital, Raipur, Chhattisgarh, India *Email: drnobalchandrakar@gmail.com

**Keywords:** Abscess, leprosy, ulnar nerve, magnetic resonance imaging, ultrasonography

## Abstract

**Introduction:**

Segmental necrotizing granulomatous neuritis (SNGN) is a rare complication of leprosy involving peripheral nerves. It can appear alone in cases of pure neuritic leprosy or in combination with cutaneous lesions.

**Case Presentation:**

A 15-year-old female diagnosed with borderline tuberculoid leprosy who received prior multidrug therapy presented 2 years later with occasional pain and tingling sensations along the inner aspect of her right arm and forearm. Imaging findings suggested SNGN, which was corroborated by cytopathological examination. She was considered relapsed from leprosy, and multi-drug therapy and steroids were started, following which she reported a decrease in the size of the swelling along with no further deterioration of the sensorineural deficit.

**Discussion:**

SNGN, which is one of the rare complications of leprosy, can create diagnostic dilemmas as its differential diagnoses include reversal reactions, and peripheral nerve tumors (such as schwannoma and neurofibroma), which have been outlined in this article. SNGN is more likely when magnetic resonance imaging (MRI) shows a well-defined ovoid lesion with central necrosis and peripheral rim enhancement.

**Conclusion:**

The incidence of SNGN is on the rise due to multi-drug therapy. In our case, the patient developed SNGN, which was considered a relapse from leprosy, and multi-drug therapy and steroids were started, following which the patient reported a significant reduction in the size of the swelling with no further deterioration of the sensorineural deficit. Hence, an appropriate diagnosis of SNGN through ultrasonography and MRI will lead to favorable outcomes, ultimately benefiting the patient.

## Introduction

Leprosy, or Hansen’s disease, is a chronic granulomatous infection caused by Mycobacterium leprae. Due to their lower temperatures than the core body temperature, the sickness primarily affects the skin, eyes, peripheral nervous system, and testes. The disease is endemic to tropical and subtropical countries like India but is declining due to multi-drug therapy.^1^

Leprosy is unique in that, despite its medical aspects being addressed appropriately, the disease is still stigmatized leading to various psychosocial issues. Numerous disabling abnormalities are brought on by the illness. The afflicted people face significant psychosocial challenges, including divorce, unemployment, and displacement from their homes. The most prevalent psychiatric condition among these people is depression. For their well-being, early identification and treatment of psychiatric problems are essential. To address these problems and enhance these patients’ quality of life, integrated healthcare methods are required.^2^

A rare complication has been reported involving peripheral nerves in patients with leprosy called segmental necrotizing granulomatous neuritis (SNGN), which has been described in previous literature as “nerve abscess.”3 SNGN is associated with the borderline tuberculoid (BT) variety of leprosy. It can occur with skin lesions or be an isolated finding in pure neuritic leprosy. It can present as single or multiple nodules along the path of the thickened nerve. As the disease can be managed with early recognition and prompt treatment, appropriate knowledge of the SNGN imaging findings can help in its early identification.

Informed written consent was obtained from the patient, and the assent was obtained from the patient’s parents to publish this report and the accompanying images.

## Case Presentation

**Figure 1.**
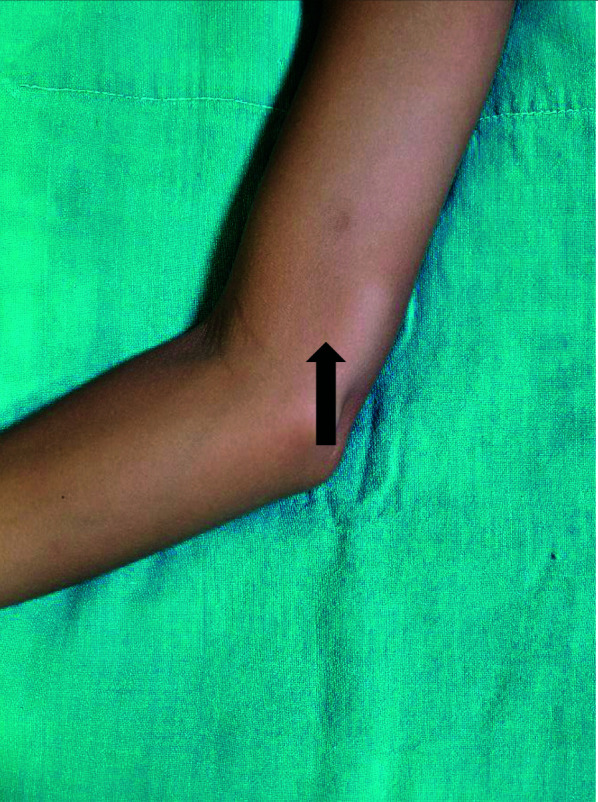
Nodular swelling (thick black arrow) of size 1 × 2 cm noted along the inner aspect of the right distal medial arm.

A 15-year-old female diagnosed with BT Leprosy who had been treated with multi-drug therapy 2 years ago presented in 2022 to the outpatient department with occasional pain and tingling sensations along the inner aspect of her right arm and forearm for 6 months, which were not associated with a fever. A soft, nodular swelling of size 1 × 2 cm was also seen at the inner aspect of the distal right arm (Figure 1). There were no hypopigmented patches noted.

**Figure 2.**
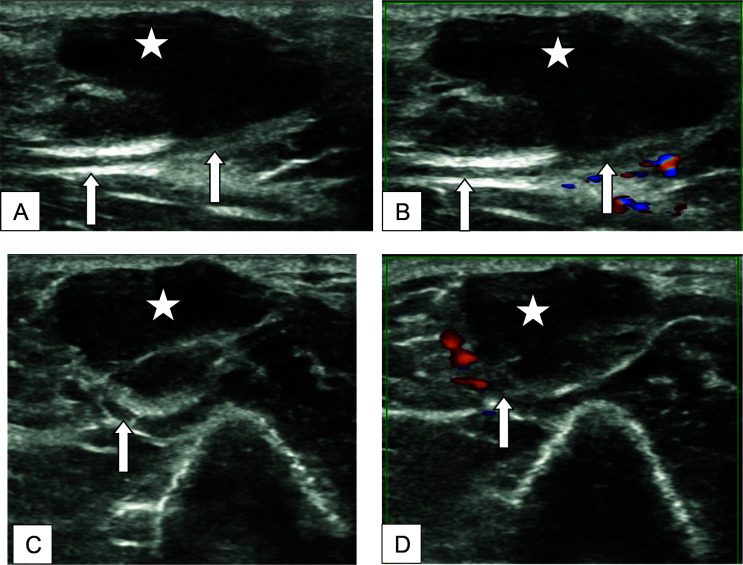
(A) Longitudinal gray scale, (B) Longitudinal color Doppler, (C) Axial gray scale, and (D) Axial color Doppler ultrasound images. Well-defined irregular-shaped hypoechoic lesion (star) with internal debris noted adjacent to the ulnar nerve (thick white arrows), not showing vascularity on color Doppler study.

On ultrasonography (USG) (Figures 2A–D), there was a well-defined irregular-shaped hypoechoic lesion with internal debris noted adjacent to the ulnar nerve on the inner aspect of the distal right arm, measuring approximately 1.0(AP) × 1.2(TR) × 3.1(CC) cm. The lesion did not show vascularity on the color Doppler study.

**Figure 3.**
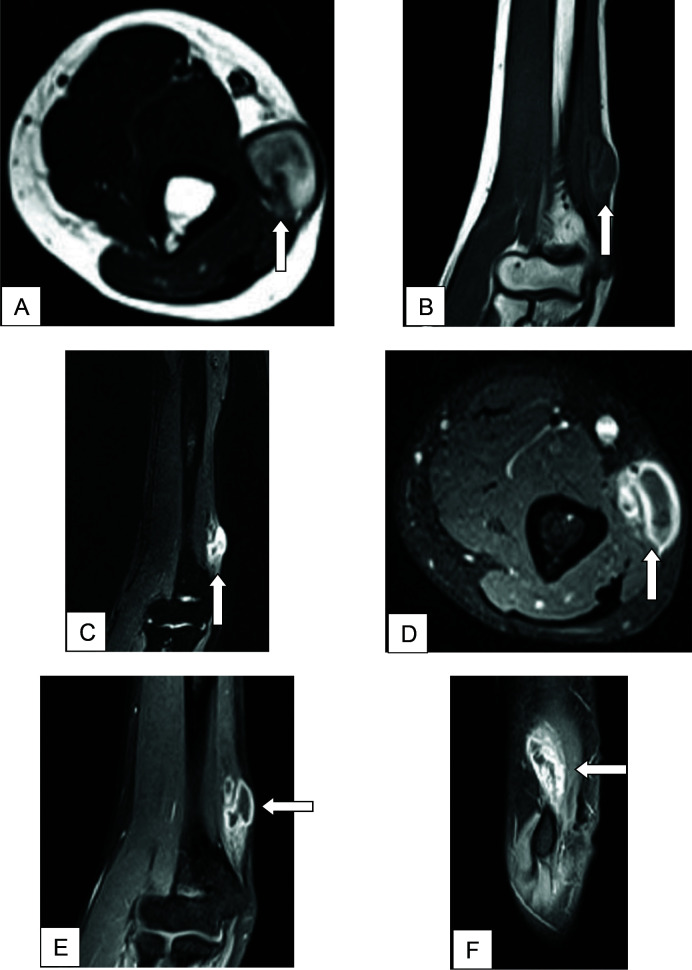
(A) Axial T2-weighted, (B) Coronal T1-weighted, (C) Coronal STIR, (D) Axial fat-suppressed T1-weighted post-contrast, (E) Coronal fat-suppressed T1-weighted post-contrast, and (F) Sagittal fat-suppressed T1-weighted post-contrast MRI images. T1 isointense, T2/STIR hyperintense lesion (thick white arrows) noted in the medial aspect of the distal right arm in the subcutaneous plane showing peripheral rim enhancement on T1 post-contrast scan.

On magnetic resonance imaging (MRI) (Figures 3A–F), an irregular, well-defined, T1 isointense, T2/STIR hyperintense lesion was noted in the medial aspect of the distal end of the right arm in the subcutaneous plane adjacent to the ulnar nerve, showing peripheral rim enhancement on the T1 post-contrast scan, likely arising from the ulnar nerve. The lesion measured approximately 1.4(AP) × 1.8(TR) × 3.5(CC) cm.

**Figure 4.**
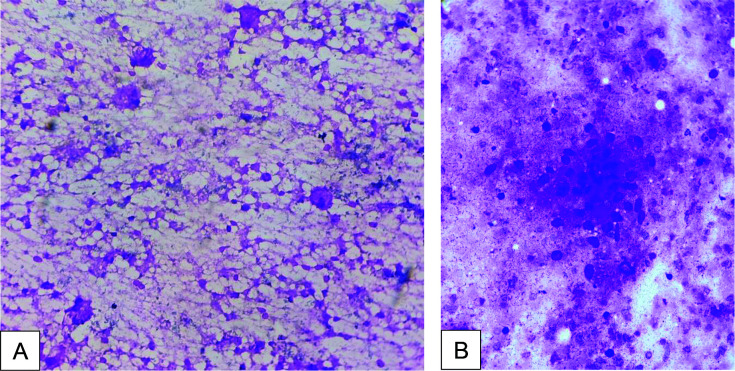
(A)&(B): Cytopathological examination. Collection of epithelioid cells, lymphocytes, plasma cells and cyst macrophages with necrotic background.

The patient was followed up with a cytopathological examination of the swelling, which showed a collection of epithelioid cells, lymphocytes, plasma cells, and cyst macrophages with a necrotic background (Figures 4A, B). Oval-to-spindle cells with pointed ends were occasionally dispersed and clustered in pinkish fibrillary matrix material. It was negative for acid-fast bacilli.

The patient was considered relapsed from leprosy, and multi-drug therapy consisting of rifampin (600 mg/month) and dapsone (100 mg daily) for 6 months along with prednisolone 0.5 mg/kg was started. Corticosteroids were gradually tapered and discontinued after 3 months. The patient was followed up monthly for 6 months until the completion of multi-drug therapy. The swelling reduced significantly and ultimately subsided; hence, the patient was managed conservatively. There was also no further deterioration of the sensorineural deficit. Physiotherapy was advised for the management of sensory and motor deficits that had already occurred.

## Discussion

Leprosy is still endemic in certain parts of the world; hence, in this article, we have focused on the imaging characteristics of one of the rare complications of leprosy, SNGN, which can create diagnostic dilemmas and lead to the late diagnosis of an easily treatable disease.

Relapse in leprosy is quite a common phenomenon. Important risk factors for relapse include lepromin negative, multiple skin lesions/thickened nerves, inadequate/irregular therapy, and the presence of “persister” bacilli.^4^ In our patient, she had a few hypopigmented skin lesions without nerve thickening initially. She was treated with a paucibacillary leprosy multi-drug treatment regimen, which she completed entirely, and the skin lesions were fully resolved, following which the relapse occurred in the form of ulnar SNGN. Accordingly, our patient’s relapse may have been influenced by the existence of “persister” bacilli.

Muiri, in 1924, first described a rare complication of leprosy involving peripheral nerves presenting as a nodular swelling, which was called a “nerve abscess.”5 Later, Chandi et al. demonstrated that these lesions consist of a caseous necrotic core surrounded by epithelioid cell granulomas rather than neutrophils as seen in an abscess; hence, the term SNGN was proposed by them for this entity.^3^

In our case, we observed that the patient was of the BT type, which is more commonly associated with SNGN.^3^ As documented in previous studies, the ulnar nerve is more commonly involved in SNGN, which was also seen in our case.^1^

On ultrasound, in our case, we observed a hypoechoic lesion with internal debris adjacent to the ulnar nerve with no internal vascularity. The normal peripheral nerves show a fascicular pattern (multiple hypoechoic neuronal fascicles running parallelly against a hyperechoic background).^6^ As documented in previous literature, the nerves affected by SNGN, or nerve abscesses, show hypoechoic focal lesions with no internal vascularity.^6,7^

On MRI of our case, a T2/STIR hyperintense lesion was noted in the subcutaneous plane adjacent to the ulnar nerve, showing peripheral rim enhancement on the T1 post-contrast scan. Very little literature is available about MRI findings in peripheral neuropathy in leprosy. Edema and swelling of the affected nerve on an MRI can be one of the findings. On T1-weighted images, nerve abscesses, or SNGN, are hypointense; on T2-weighted images, they are hyperintense; and on post-contrast studies, they exhibit peripheral enhancement.^8,9^

In this instance, SNGN, reversal reactions, and peripheral nerve tumors (such as schwannoma and neurofibroma) were among the differential diagnoses. SNGN presents as swelling along the nerve and is generally associated with neurologic manifestations. SNGN is more likely when an MRI shows a well-defined ovoid lesion with central necrosis and peripheral rim enhancement. A peripheral nerve tumor presents as soft tissue swelling with no obvious neurologic manifestations. A peripheral nerve tumor shows a fusiform-shaped mass with tapered ends, appearing heterogeneously hypoechoic with internal vascularity on USG, and high signal intensity lesions showing intense homogeneous enhancement on MRI.^10,11^ Clinically, reversal responses are defined by heightened inflammation of pre-existing lesions. Nerves can thicken and become painful, and symptoms of a pre-existing peripheral neuropathy (sensory, motor, or autonomic) may appear. The USG reversal reaction shows endoneural color flow signals and Gd enhancement on MRI.^12^ Isolated findings of nerve thickening should also raise suspicion for various hypertrophic neuropathies and other entities such as Charcot-Marie-Tooth disease, Guillain-Barré syndrome, acromegaly, amyloidosis, chronic inflammatory demyelinating polyradiculoneuropathy, Dejerine-Sottas syndrome, and neurofibromatosis.^7^ Nerve thickening can also be seen in cases of repetitive trauma and nerve dislocation.^7^

The treatment options available for SNGN are multi-drug therapy, corticosteroids, and surgery. The surgery is indicated when there is a requirement for high dosages of steroids, further deterioration of neurological manifestations, and pain not controlled by steroids. The surgical option utilized involves exploration and drainage of the abscess. In our case, the patient responded well to multi-drug therapy and steroids; therefore, she was treated conservatively.^13,14^

## Conclusion

Leprosy is one of the major causes of peripheral neuropathy in endemic areas. Leprosy should always be kept in mind when a patient presents with nerve thickening and impaired sensation, as the disease can be easily cured with early diagnosis and prompt treatment before developing mutilating complications. In our case, the patient developed SNGN, which was considered a relapse from leprosy, and multi-drug therapy and steroids were started, following which the patient reported a significant reduction in the size of the swelling with no further deterioration of the sensorineural deficit. The incidence of SNGN is on the rise due to multi-drug therapy. Hence, an appropriate diagnosis of SNGN through USG and MRI will lead to favorable outcomes, ultimately benefiting the patient.

## Data Availability Statement

It is a case report, and all data, including images, are being submitted in the case report itself.

## Authors’ Contributions

All authors analyzed and interpreted the patient data, collected the imaging, and wrote the manuscript.

## Consent for Publication

Informed written consent was obtained from the patient and the patient’s parent to publish this report and the accompanying images.

## Competing Interests

The authors declare that there are no competing interests.
